# Management of oculo-orbital complications of odontogenic sinusitis in adults

**DOI:** 10.22336/rjo.2024.09

**Published:** 2024

**Authors:** Mihai Alexandru Preda, Codruț Sarafoleanu, Gabriela Mușat, Andreea-Alexandra Preda, Daniel Lupoi, Ramona Barac, Monica Pop

**Affiliations:** * “Carol Davila” University of Medicine and Pharmacy, Bucharest, Romania; ** ENT Department, “Sf. Maria” Clinical Hospital, Bucharest, Romania; *** Ophthalmology Department, Clinical Emergency Eye Hospital, Bucharest, Romania; **** Rheumatology Department, “Sf. Maria” Clinical Hospital, Bucharest, Romania

**Keywords:** odontogenic maxillary sinusitis, ophthalmological complications, multidisciplinary management

## Abstract

**Introduction:** Odontogenic maxillary sinusitis (OMS) is an infectious inflammatory pathology caused by a dental condition. Considering the anatomical relations with the orbit, maxillary sinus infection can easily spread, evolving into severe oculo-orbital complications that can sometimes be life-threatening.

**Material and methods:** We performed a retrospective study of over 2 years, examining the data of 18 patients diagnosed with OMS with oculo-orbital complications. The patients were evaluated regarding their dental history, symptoms, clinical and endoscopic findings, ophthalmologic evaluation, bacteriologic tests, computed tomography (CT) imaging, medical and surgical treatment, and outcomes.

**Results:** The age of the patients was between 24 and 65 years old with an almost equal gender distribution: 10 female and 8 male patients. From the total, 7 patients had type II diabetes, 2 of whom were insulin-dependent, 1 patient had thrombophilia and 2 patients had renal failure with peritoneal dialysis. Regarding the type of oculo-orbital complications, 10 patients were diagnosed with preseptal cellulitis and 8 with orbital cellulitis. Just 5 patients with orbital cellulitis required surgical treatment and orbitotomy was performed, followed by endonasal endoscopic drainage. The evolution after surgical treatment was favorable for all operated patients.

**Discussions:** Oculo-orbital complications of OMS are typically more severe than those of rhinogenic sinusitis because anaerobic bacteria are involved. Immunosuppression represents a favorable environment for the development of OMS and its complications, diabetes being the most common risk factor. A negative prognostic feature is the appearance of ophthalmological symptoms in both eyes, so visual function may be reduced. The treatment of oculo-orbital complications of OMS is urgent and depends on a broad-spectrum antibiotic therapy associated or not with surgical intervention.

**Conclusions:** The diagnosis of oculo-orbital complications of OMS is complex and requires clinical experience as well as extensive medical knowledge to treat both the cause and the consequences of the conditions quickly and effectively. The proper management of oculo-orbital complications is based on a multidisciplinary team: ophthalmology, ENT, dentistry, imaging, and laboratory.

**Abbreviations:** OMS = odontogenic maxillary sinusitis, CT = computed tomography, ENT = ear-nose-throat, MRI = magnetic resonance imaging, HNS = head and neck surgery

## Introduction

Chronic odontogenic sinusitis is defined as an infectious-inflammatory rhinosinusal pathology with a dental starting point. In the context of the development of dental techniques together with the higher addressability to dental services, sinusitis of odontogenic etiology is becoming increasingly frequent [**1**,**2**]. Recent research data have shown an incidence of OMS of up to 40% of all chronic maxillary rhinosinusitis, which represents a striking contrast compared to 10 years ago when the incidence was only 10-12% [**3**-**5**]. 

This pathology implies an increased risk of developing major complications, some of which are life-threatening. To avoid such situations, prophylactic education is required in the sense of periodic evaluations and early presentation to a specialist. In this way, a correct management of the disease and an individualized and complete therapeutic plan can be established [**6**,**7**]. 

The description of maxillary sinus oculo-orbital complications is based on the Chandler classification system [**8**]. This classification includes five stages:

1. preseptal cellulitis: inflammation and edema anterior to the orbital septum;

2. orbital cellulitis: inflammation and edema posterior to the orbital septum;

3. subperiosteal abscess between the lamina papyracea and the orbital periosteum;

4. orbital abscess;

5. cavernous sinus thrombosis.

The simple oculo-orbital complications of odontogenic sinusitis in adults can initially manifest by persistent conjunctival hyperemia and not be influenced by the treatments of instillation of antibiotic eye drops, oral antibiotics, corticosteroids, and/or non-steroidal anti-inflammatory drugs. Unfortunately, patients frequently self-treat, which causes the alteration of the local flora, the delay of the etiological diagnosis, and thus the progression of the condition. In the case of persistent conjunctival hyperemia or recurrences, the ophthalmologist must initiate additional investigations but not delay by replacing a local antibiotic-steroid treatment. Of course, before starting the treatment, conjunctival secretion samples are necessary for bacteriological tests. In everyday practice, this step is frequently avoided [**9**,**10**]. 

More advanced oculo-orbital complications of odontogenic sinusitis in adults include anterior, posterior, or anteroposterior uveitis [**11**]. In anterior uveitis, patients present for changes in visual acuity, and the ophthalmological clinical examination reveals perikeratic hyperemia, endothelial precipitates, irionic synechiae, and exudation in the anterior chamber. In the case of posterior uveitis, the changes are profound, affecting the vitreous or even the vitreoretinal surface. They are associated with a marked decrease in visual acuity [**12**,**13**]. In these increasingly frequent situations, in addition to complete laboratory investigations and after excluding other causes of uveitis, the ENT and dental examination are important for the detection of possible sinus and dental infections, along with their treatment. 

*Periorbital cellulitis*

Preseptal cellulitis or periorbital cellulitis represents the infection of the skin and soft tissue localized around the eye, anterior to the orbital septum. Usually, patients present to the specialist with unilateral eyelid edema and erythema and it rarely causes severe complications if the appropriate treatment is immediately instituted. The diagnosis also requires a CT scan of the orbits and sinuses. Antibiotic treatment, both local and systemic, anti-inflammatory and anticoagulant medication is necessary, all these simultaneously with the treatment of the generating infection. Of course, a thorough anamnesis is required, with concrete data, to be able to treat in the context of possible comorbidities and to avoid negative drug interactions [**14**-**17**]. 

*Orbital cellulitis*

Orbital cellulitis, also referred to as postseptal cellulitis is one of the most severe complications, requiring urgent treatment. It represents a severe infection of the muscle and the soft tissue located within the orbit, without involving the globe. The clinical presentation is similar to preseptal cellulitis, with the addition of ophthalmoplegia with diplopia, eye movement pain, and/or proptosis. Chemosis is also present, as in the case of severe periorbital cellulitis [**18**,**19**]. The diagnosis of orbital cellulitis must be certain and can be confirmed with imaging (CT or MRI), to immediately initiate antibiotic, anticoagulant and supportive treatment. In some serious cases, surgery may be required. The efficient treatment is mandatory, considering that this condition may have serious complications including loss of vision, abscesses (subperiosteal or orbital), intracranial extension of the infection, or even cavernous sinus thrombosis. The bacteriological findings are commonly bacterial and can include both aerobic and anaerobic germs. Rarely, fungi or mycobacteria can cause orbital cellulitis. In any case, this complication is an ocular emergency that requires the collaboration of a medical team including an ophthalmologist, an ENT doctor, a radiologist, and sometimes an infectious disease specialist or a cardiologist [**14**,**15**,**19**,**20**]. 

*Cavernous sinus thrombosis*

Cavernous sinus thrombosis is a life-threatening condition that can result in a complication of facial infections, orbital cellulitis, sinusitis, pharyngitis, or otitis, or in the case of traumatic injuries or surgery [**21**]. Clinically, it presents with headache, fever, and ocular symptoms such as periorbital edema and pain, photophobia, diplopia, loss of vision, or ophthalmoplegia. The early recognition of this complication is essential, even lifesaving. The diagnosis is based on clinical aspects as well as imaging with either a contrast-enhanced CT scan or MRI scan [**22**,**23**]. Although it is not a common condition and there are no precise guidelines regarding its management, first-line treatment should include antimicrobial (anti-staphylococcal antibiotics, a third-generation cephalosporin, metronidazole for anaerobes and amphotericin B as antifungal) and anticoagulant therapies (unfractionated heparin or low molecular weight heparin) [**23**-**25**]. Regardless of maximal treatment, the risk of long-term sequelae such as vision loss, diplopia, and stroke remains significant [**23**].

Immunosuppression is one of the risk factors for the development of these complications, as well as some serious, chronic, or acute conditions of the affected patients. Such a context accompanied by the postponement of the presentation to the ENT specialist and/or dentist for any sinus or dental inflammatory process, will pave the way for its propagation to the adjacent tissues.

## Materials and methods

This study aimed to clarify and elaborate on the key points in the management of odontogenic maxillary sinusitis with oculo-orbital complications. 

We performed a retrospective clinical study of over 2 years, between January 2020 and December 2022, examining the data of 18 patients diagnosed with OMS with oculo-orbital complications, admitted to the ENT HNS Clinic of “Sf. Maria” Hospital in Bucharest. 

The patients’ data were obtained from the observation files, following the next inclusion criteria: adult patients over 18 years old, clinically and paraclinically confirmed diagnosis of OMS with oculo-orbital complications, patients who gave their consent to participate in the study. 

We evaluated the patients regarding their history, symptoms, clinical and endoscopic evaluation, ophthalmologic involvement, ophthalmologic evaluation, bacteriologic cultures, CT imaging, dental involvement, medical and surgical treatment, and outcomes. 

Regarding the symptoms, for the diagnosis of sinusitis, they were divided as follows:

• Major criteria including pain, the sensation of fullness or facial pressure, nasal obstruction, anterior or posterior rhinorrhea or purulent rhinorrhea in anamnestic/clinical examination, cacosmia, fever;

• Minor criteria including headache, fever (from other cause than acute rhinosinusitis), halitosis, fatigue, toothache, cough, otalgia, or ear pressure sensation;

Therefore, to support the diagnosis of rhinosinusitis, patients had to present two major signs/symptoms or one major sign/symptom and two minor ones.

From the anterior rhinoscopy evaluation, we noted the aspect of the nasal mucosa, the presence of anatomical variations (nasal septum deviations, inferior nasal turbinates hypertrophy, pneumatization of the middle nasal turbinates), as well as the presence of mucopurulent secretions in the middle nasal meatus.

During nasal endoscopy, the following aspects were evaluated: the condition of the middle nasal meatus, the nasopharynx, and the mucociliary drainage paths. Also, the endoscopic nasal examination was useful for sampling rhinosinusal secretions for bacteriological and mycological examinations, as well as preoperatively in the study of anatomical variants and sinus drainage, to avoid intraoperative complications. 

Regarding imaging investigations, a CT scan, the gold standard in the diagnosis of rhinosinusitis, was performed on all patients. Completed in all 3 planes (axial, coronal, and sagittal), with a variable thickness, this investigation offered a high-quality spatial resolution, with the visualization of the osteomeatal complex and the relationships with the orbit and base of the skull. 

Definitive diagnosis was based on clinical and endoscopic ENT examination, CT scan of the sinuses, and dental examination showing underlying dental pathology. The ophthalmologist evaluated the oculo-orbital complications both clinically and imagistically.

## Results

**Fig. 1:**
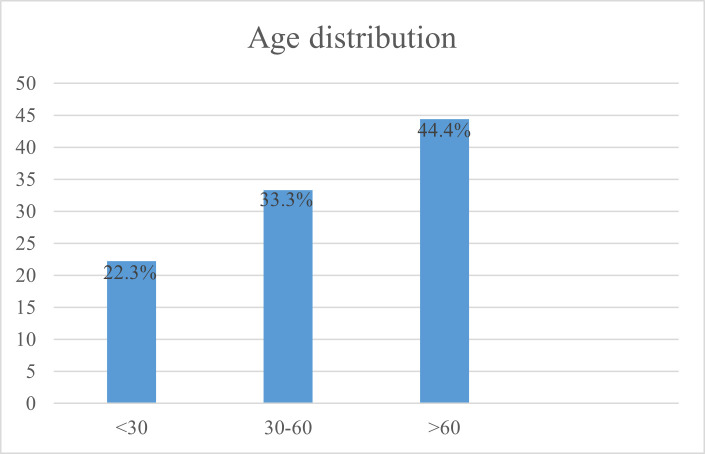
Distribution of patients by age group

The age of the patients in this study was between 24 and 65 years old with an almost equal gender distribution: 10 female and 8 male patients. Regarding the distribution of patients by age group, 4 patients were under 30 years old, 6 patients between 30-60 years old, and 8 patients over 60 years old (**Fig. 1**). 

**Fig. 2:**
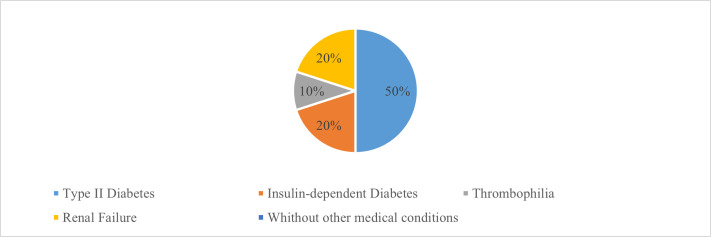
Risk factors for developing oculo-orbital complications

The risk factors encountered in the studied group were distributed as follows: 7 patients had type II diabetes, 2 of whom were insulin-dependent, 1 patient had thrombophilia and 2 patients had renal failure with peritoneal dialysis. The remaining 8 patients did not have other medical conditions apart from the dentoalveolar pathology causing the maxillary sinusitis (**Fig. 2**).

Of the 18 patients, all initially had neglected ophthalmological and/or dental symptoms.

After an ENT examination, complex laboratory investigations, and imaging, a common etiology of odontogenic nature was found.

Regarding the symptoms, the following were highlighted: low fever in 7 patients and temperature above 38° in 6 patients. The rest of the patients were afebrile.

**Fig. 3:**
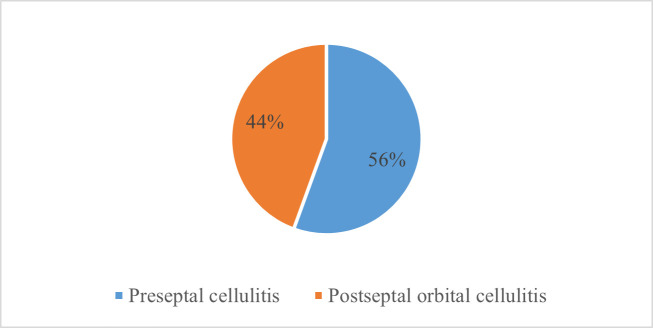
Oculo-orbital complications of OMS

10 patients were diagnosed with preseptal cellulitis and 8 with orbital cellulitis (**Fig. 3**).

The bacteriological examination of the nasopharynx revealed Streptococcus aureus, Gram-negative cocci in 14 patients and, in 4 patients, Aspergillus as the etiological agent. 

The examination performed by the ENT specialist, combined with the ophthalmological, dental, and imaging examinations, indicated the direction toward a correct etiological diagnosis and an appropriate therapeutic attitude. Sustained emergency treatment, with combined parenteral antibiotic therapy, among antifungal, anti-inflammatory, and anticoagulant treatment was instituted, along with the treatment for chronic conditions in the case of patients with comorbidities. Also, the dentist or the oral and maxillofacial surgeon eradicated the underlying dental pathology that created the odontogenic sinusitis and consequently the oculo-orbital complications. Moreover, the ophthalmologist instituted local treatment of the eye. 

Five patients with orbital cellulitis required surgical treatment, orbitotomy followed by endonasal endoscopic drainage being performed. The evolution after surgical intervention was favorable in five operated patients, and slower in two of those with comorbidities (diabetes).

The number of hospitalization days was between 9-14, the patients being discharged while healing, and 21 days in the case of one patient who received dialysis and another one with type I diabetes.

## Discussion

Oculo-orbital complications are one of the most frequent complications of rhinosinusitis, representing around 80% of the cases [**26**,**27**]. Because anaerobic bacteria are involved, oculo-orbital complications of odontogenic sinusitis are typically more severe than those of rhinogenic sinusitis [**28**]. Aspects regarding which gender is most affected by these complications are largely discussed in the specialized literature. Most authors [**26**,**29**,**30**] noted that ocular problems from acute sinusitis were more common in men. On the other hand, female predominance was noted in the series described by Ben Ammor [**31**]. Our study was non-discriminatory from this point of view, the gender distribution being balanced, with 10 female patients (55.55%) and 8 male patients (44.45%). 

Immunosuppression provides a favorable environment for acute sinusitis to develop [**32**]. One of the most well-known immunosuppressive factors and a major risk factor for cavernous sinus thrombosis is diabetes [**33**]. Immunosuppression can also be caused by HIV infection [**32**], medullary aplasia following chemotherapy, radiation therapy [**27**,**30**,**31**], issues with local nasal immunity, and hypogammaglobulinemia. Our study confirmed the data from the literature, meaning that more than half of our patients had a favorable immunosuppressive factor. Thus, 7 patients (38,8%) had diabetes type 2, of whom 2 patients (11,11%) had insulin-dependent diabetes, 2 patients had renal insufficiency with peritoneal dialysis, and 1 patient (5,55%) had thrombophilia. The rest of the patients (44,45%) did not have any other diseases, except the dentoalveolar pathology that generated the odontogenic sinusitis. 

Regarding the affected part by the oculo-orbital complications of the odontogenic sinusitis, all the patients in the study had ipsilateral dental pathology. A negative prognostic feature is the appearance of ophthalmological symptoms in both eyes, so visual function may be reduced [**34**]. Research findings indicate that odontogenic rhinosinusitis is more likely to cause visual impairment than rhinogenic sinusitis [**7**]. Not a single patient in our study experienced visual acuity loss. This could be because all the patients received early antibiotic treatment along with anaerobic coverage and adequate surgical therapy.

The oculo-orbital consequences of rhinosinusitis are typically caused by ethmoidal rhinosinusitis [**6**], but odontogenic maxillary sinusitis extends to the orbit more commonly than predicted. Preseptal cellulitis was the most commonly reported problem in the majority of studies [**30**,**35**], but according to a Tunisian study [**36**], approximately half of the patients had subperiosteal abscesses. Our study showed similar results: 10 of the patients (55,55%) were diagnosed with preseptal cellulitis, while 44,45% had postseptal cellulitis. 

Literature findings highlight the prevalence of anaerobes, specifically Porphyromonas, Peptostreptococcus, Prevotella, and Fusobacterium, among the bacteria involved in the physiopathology of odontogenic sinusitis. However, aerobes of both Gram-positive and Gram-negative types can also be found in the cultures [**37**]. Odontogenic rhinosinusitis is thought to be a polymicrobial infection that primarily consists of anaerobes and gathers germs from the respiratory system and mouth cavity [**38**]. The bacteriological characteristics of odontogenic and rhinogenic sinusitis differ significantly [**39**]. The most typical finding in fungal sinusitis is aspergillosis of the maxillary sinus on a foreign body with dental origins [**31**,**40**]. The nasopharyngeal cultures collected from our patients highlighted the presence of bacteria such as Staphylococcus aureus, gram-negative cocci, or fungi like Aspergillus in 4 patients. 

The introduction of new antibiotic compounds has greatly reduced the occurrence of these life-threatening complications [**26**,**41**]. The treatment of oculo-orbital complications from OMS is urgent and depends on a broad-spectrum antibiotic therapy that is active against the predominant aerobic and anaerobic bacteria in the foreground [**42**,**43**], associated or not with a surgical intervention [**26**,**43**]. After the bacteriological results have been obtained, the antibiotic treatment must be targeted according to the antibiogram [**41**,**43**]. Our patients received sustained emergency treatment, with combined parenteral antibiotic therapy, among antifungal, anti-inflammatory, and anticoagulant treatments. They also received treatment for chronic conditions in the case of comorbidities. Also, the dentist or the oral and maxillofacial surgeon eradicated the underlying dental pathology that created the odontogenic sinusitis and consequently the oculo-orbital complications. Moreover, the ophthalmologist instituted local treatment of the eye. Before the clinical exacerbation under treatment, surgical orbital drainage is recommended [**41**]. It needs to be done quickly to prevent major side effects like compression of the optic nerve or cavernous sinus thrombosis [**33**,**44**]. The next step is functional endoscopic sinus surgery (FESS), to improve sinus ventilation and drainage and eradicate the inflammatory disease that results in drainage tract occlusion [**31**]. The two main goals of sinus surgery are the establishment of a broad channel of communication between nasal pits and sinuses, as well as the preservation of anatomical landmarks and respiratory mucosa. Five of our patients with postseptal cellulitis needed surgical treatment. They suffered orbitotomy, followed by endonasal endoscopic drainage. It is important to emphasize that ocular sequelae cannot always be avoided, not even with an aggressive and early surgical intervention. According to Harris [**45**] and Spires [**46**], the incidence of sequelae blindness is 14% and 33%, respectively. 

Under medical care, preseptal cellulitis and sub-periosteal abscesses frequently develop favorably and do not cause any problems [**34**,**42**]. Nevertheless, occasionally, in cases with sub-periosteal abscess, a clinical exacerbation (a decrease in visual acuity) can be observed. In the cases of the 5 patients previously mentioned, their postoperative evolution was favorable, and slower in 2 patients with diabetes.

Appropriate treatment planning is the first step towards preventing these complications. A comprehensive clinical examination, a full medical history, and imaging exams should be all part of a correct diagnostic algorithm. A history of sinus disease or any surgical interventions, as well as the presence of a tendency to develop upper respiratory tract pathologies, must be obtained from the anamnesis to complete the medical history.

## Conclusions

The diagnosis of oculo-orbital complications of OMS is complex and requires clinical experience as well as extensive medical knowledge to treat both the cause and the consequences of the conditions quickly and effectively. The correct and complete management of oculo-orbital complications is based on a multidisciplinary team: ophthalmology, ENT, dentistry, imaging, and laboratory. Associated comorbidities are risk factors for the appearance of this kind of complication. A certain and fast diagnosis, as well as the initiation of an appropriate, often extensive treatment, is necessary for the favorable evolution of the disease and the prevention of relapses. Most patients were cured under long-term treatment and strict monitoring. Education and periodic check-ups are important factors in preventing severe or very severe complications of odontogenic sinusitis, some of which are life-threatening in the absence of an early and adequate treatment. 

**Conflict of Interest Statement**

The authors state no conflict of interest. 

**Informed Consent and Human and Animal Rights Statement**

Informed consent has been obtained from all individuals included in this study. 

**Authorization for the use of human subjects**

Ethical approval: The research related to human use complies with all the relevant national regulations and institutional policies, is by the tenets of the Helsinki Declaration, and has been approved by the review board of “Sf. Maria” Clinical Hospital, Bucharest, Romania (05.12.2023/30011).

**Acknowledgments**

None. 

**Sources of Funding**

None. 

**Disclosures**

None. 

**Competing interests**

None.
